# From dietary exposure to organ injury: microbiome-mediated biotransformation of food-chain xenobiotics across the gut–liver–kidney axis

**DOI:** 10.3389/fmicb.2026.1977638

**Published:** 2026-10-13

**Authors:** Guolin Li, Yu Xiong, Xixuan Yu, Jiangping Yu, Ruiling Xu

**Affiliations:** 1 Department of Pharmacy, Mianyang Central Hospital, School of Medicine, University of Electronic Science and Technology of China, Mianyang, China; 2 Department of Pharmacy, Sichuan First Hospital of Traditional Chinese Medicine, Chengdu, Sichuan, China; 3 NHC Key Laboratory of Nuclear Technology Medical Transformation, Mianyang Central Hospital, School of Medicine, University of Electronic Science and Technology of China, Mianyang, China

**Keywords:** chronic kidney disease, food-chain xenobiotics, gut microbiota, gut-liver-kidney axis, metabolic dysfunction-associated steatotic liver disease, microbial biotransformation

## Abstract

Food-chain xenobiotics reach the intestine through food additives, processing-generated chemicals, environmental contaminants, and naturally occurring toxins. Their biological effects depend not only on intake but also on microbial metabolism and host handling. Gut microorganisms can detoxify parent compounds or generate metabolites with altered biological activity, while xenobiotic exposure can reshape microbial communities and host–microbial interactions. This review synthesizes evidence on these processes across the gut–liver–kidney axis, with particular attention to metabolic dysfunction-associated steatotic liver disease and chronic kidney disease. We examine direct microbial biotransformation alongside exposure-related changes in intestinal barrier function, immune responses, and metabolic signaling. The resulting synthesis highlights compound-specific and host-specific responses, shaped by microbial enzyme capacity, dietary context, barrier integrity, and hepatic and renal clearance. Impaired organ function can, in turn, alter microbial ecology and promote metabolite accumulation, creating bidirectional interactions between exposure and disease. Although experimental studies provide mechanistic insights, human evidence establishing causal links between dietary xenobiotics, microbial transformation, and chronic liver or kidney injury remains limited. We also evaluate the potential of integrated biomarkers, dietary interventions, and microbial enzyme-targeting strategies, identifying the evidence needed for clinical translation. Future progress requires longitudinal cohorts and controlled dietary studies that combine measured exposure, microbial function, metabolite profiles, and organ-specific outcomes. This integrated perspective provides a framework for investigating individual susceptibility to dietary xenobiotics and evaluating microbiome-informed approaches to food safety and nutrition.

## 1 Introduction

Modern diets expose consumers to xenobiotic chemicals from several food-chain sources. These include nitrosamines, heterocyclic amines (HCAs) and polycyclic aromatic hydrocarbons (PAHs) generated during high-temperature processing or cooking. They also include additives, contaminants and other processing-related exposure components. Evidence links these exposures to intestinal genotoxicity, mucosal injury and microbial dysbiosis, and to higher risks of obesity, lipid dysregulation, impaired glucose metabolism and fatty liver disease (Nogacka et al., 2019; Saha and Samuel, 2024; Barra et al., 2025).

The gut microbiota is a central interface between dietary xenobiotic exposure and host phenotype. Microorganisms can reduce, deconjugate, cleave and further modify xenobiotics, thereby changing bioavailability, toxicity and elimination routes. Xenobiotics can also reshape microbial composition and select strains with degradation or tolerance capacity (Swanson, 2015). Classic experimental work showed that the human gut microbiota chemically transforms xenobiotic compounds (Koppel et al., 2017). More recent studies extended this principle to dietary xenobiotics and environmental pollutants, showing detoxification, toxification and enrichment of xenobiotic-degradation functions in human-associated microbiomes (Culp et al., 2024; De Filippis et al., 2024). Gut microbes may also influence host drug-metabolizing enzymes, transporters and nuclear receptor pathways, which can buffer or amplify exposure effects (Claus et al., 2016; Chiu et al., 2020; Chi et al., 2021).

The gut-liver-kidney axis is useful because orally encountered xenobiotics rarely act within one compartment. Gut-derived metabolites, inflammatory mediators and bacterial components can reach the liver and kidney through portal and systemic circulation. Conversely, hepatic or renal impairment can alter xenobiotic clearance and redistribution, creating feedback between exposure, metabolism and organ function. A recent gut-liver-kidney axis study emphasized this metabolic and immunological coupling in chronic liver and kidney disease (Fei et al., 2025).

This Review therefore asks how food-chain xenobiotic exposure is modified by gut microbial metabolism, and how that modification may alter chronic disease risk along the gut-liver and gut-kidney axes. We synthesize major exposure classes, microbial enzyme systems, metabolic pathways and organ-level consequences, with attention to barrier disruption, immune activation and metabolic reprogramming (Figure 1). The aim is not to catalogue every xenobiotic, but to identify where microbial transformation changes the interpretation of dietary exposure.

**Figure 1:**
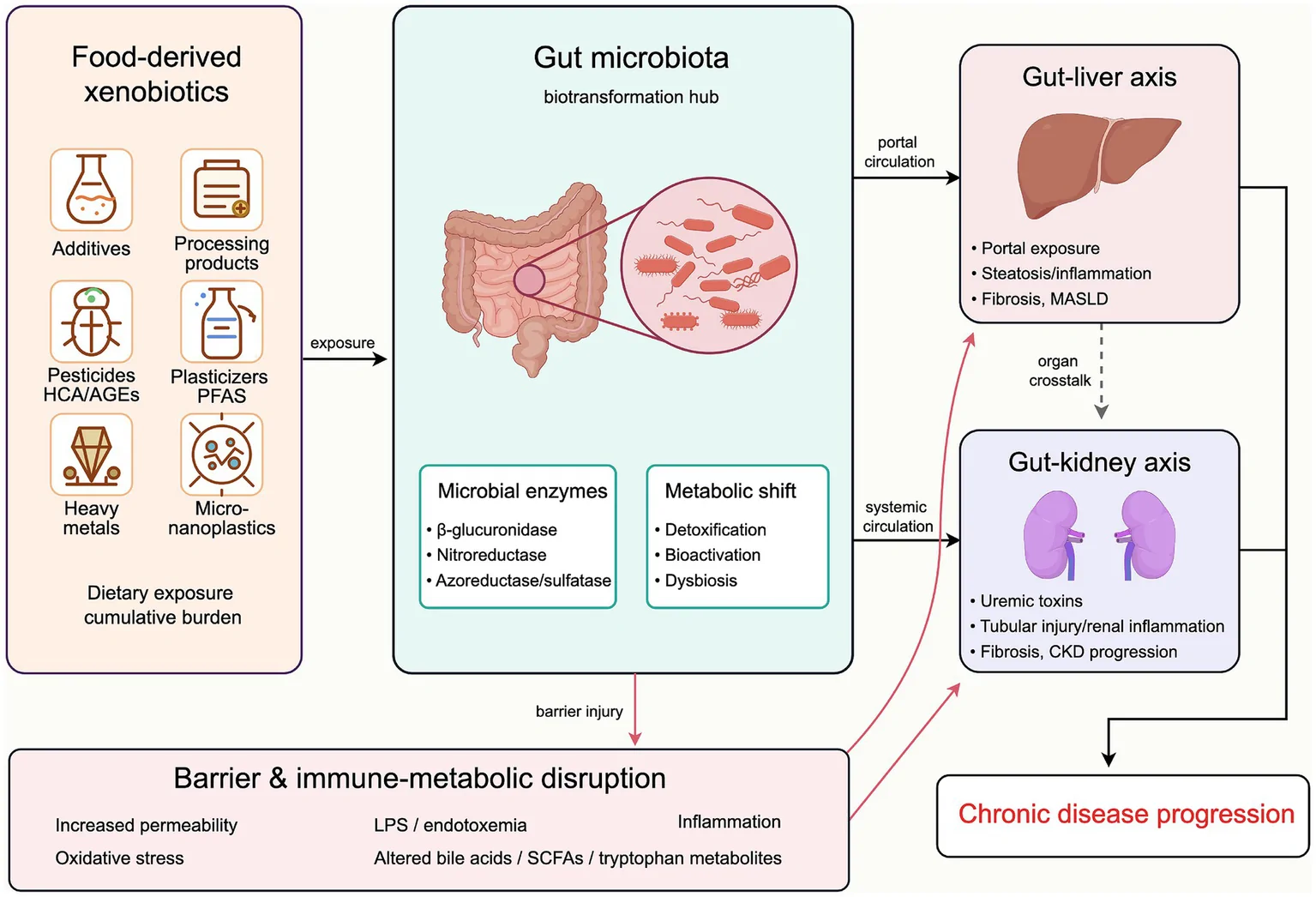
Food-derived xenobiotics, gut microbial biotransformation, and the gut–liver–kidney axis. Food-derived xenobiotics, including food additives, processing-derived compounds, pesticides, plasticizers, per- and polyfluoroalkyl substances (PFAS), heavy metals, and micro−/nanoplastics, enter the gastrointestinal tract through dietary exposure and cumulative intake. The gut microbiota acts as a central biotransformation hub, mediating xenobiotic detoxification or bioactivation through microbial enzymes such as β-glucuronidase, nitroreductase, azoreductase, and sulfatase. Xenobiotic-induced dysbiosis and altered microbial metabolism may disrupt intestinal barrier integrity and promote immune-metabolic disturbances, including increased permeability, endotoxemia, inflammation, oxidative stress, and altered microbial metabolites. These signals are transmitted to the liver through portal circulation and to the kidney through systemic circulation, contributing to hepatic steatosis, inflammation, fibrosis, tubular injury, renal inflammation, and chronic disease progression. Crosstalk between the liver and kidney may further amplify inflammatory and metabolic injury. Abbreviations: AGEs, advanced glycation end products; CKD, chronic kidney disease; HCAs, heterocyclic amines; MASLD, metabolic dysfunction-associated steatotic liver disease; PFAS, per- and polyfluoroalkyl substances; SCFAs, short-chain fatty acids.

Operationally, the term food-chain xenobiotic is used for four exposure classes: intentionally added but exogenous food additives; chemicals generated during processing or cooking; environmental contaminants transferred into food or drinking water; and naturally occurring food toxins. The label describes route and biological foreignness, not a shared toxicological potency. Polyphenols are treated separately as microbiota-transformable food bioactives, tryptophan, tyrosine, choline and carnitine as dietary metabolic precursors, and bile acids as host endobiotics modified by microbial enzymes (Chen et al., 2022b; Paik et al., 2022). These comparator classes are included only where they clarify common microbial reactions or organ-clearance constraints.

This review was based on peer-reviewed English-language literature indexed in PubMed, Web of Science and Scopus. Exposure terms included food-derived xenobiotics, food additives, emulsifiers, sweeteners, processing contaminants, mycotoxins, pesticides, per- and polyfluoroalkyl substances (PFAS), plasticizers, heavy metals and microplastics (MPs). Mechanistic and outcome terms included gut microbiota, microbial biotransformation, gut-liver axis, gut-kidney axis, metabolic dysfunction-associated steatotic liver disease (MASLD), chronic kidney disease (CKD), barrier function, bile acids, uremic toxins, metagenomics and metabolomics. Priority was given to studies with defined exposure classes, microbial or metabolic measurements and organ-relevant outcomes. Human intervention and cohort data were weighted most heavily for translational interpretation; animal, *in vitro* fermentation and omics studies were used mainly to define mechanisms. Broad microbiome associations without exposure resolution, non-peer-reviewed sources and records with incomplete bibliographic traceability were not used as primary evidence. Unlike previous reviews that focused on individual food additives, food contaminants, gut microbiota dysbiosis, or single-organ outcomes, this Review integrates food-chain xenobiotic exposure, microbial biotransformation, and gut–liver–kidney axis mechanisms into a unified food-safety and food-health framework. This perspective may help interpret why identical dietary exposures can produce different biological outcomes depending on microbial enzyme capacity, intestinal barrier status, and hepatic or renal clearance.

## 2 Food-chain sources and exposure features of dietary xenobiotics

Food additives are among the most common xenobiotic exposures in modern diets. The clearest microbiome evidence currently involves emulsifiers and non-nutritive sweeteners, which can alter microbial composition, metabolic activity and mucosal barrier function (Li P. et al., 2024; Wang H. et al., 2024). In animal and ex vivo human microbiota models, carboxymethylcellulose (CMC), polysorbate 80 (P80), lecithin, sucrose fatty acid esters and mono−/diglycerides have been linked to dysbiosis and pro-inflammatory phenotypes. Human ex vivo systems also show that commonly used emulsifiers can directly reshape gut microbial composition and function (Chassaing et al., 2015; Naimi et al., 2021). Non-nutritive sweeteners show a more individualized pattern. In a human study, sweetener effects on glucose tolerance depended strongly on baseline microbiota, indicating that the same additive can produce different metabolic responses across hosts (Suez et al., 2022). Table 1 summarizes the main food-derived xenobiotic categories, exposure routes and potential microecological effects.

**Table 1:** Food-chain xenobiotic classes and microbiota-relevant transformations.

Exposure class	Representative food-chain sources	Representative compounds	Microbial transformation or perturbation	References
Emulsifiers	Ultra-processed foods, sauces, ice cream, bakery products, processed dairy and ready-to-eat foods	CMC, P80, lecithin, sucrose fatty acid esters, mono−/diglycerides	May alter mucus-associated communities, barrier integrity, SCFA production and inflammatory tone	Chassaing et al. (2015, 2017), Naimi et al. (2021)
Non-nutritive sweeteners	Diet beverages, low-calorie desserts, sugar-free foods and tabletop sweeteners	Saccharin, sucralose, aspartame, acesulfame potassium, stevia-derived sweeteners	Microbiome-dependent shifts in carbohydrate metabolism, glycemic response and microbial metabolites	Suez et al. (2022)
Processing-generated xenobiotics	Grilled, fried, roasted, barbecued or smoked foods; baked and fried high-AGE foods	HAAs/HCAs, PAHs, nitroso compounds, CML, CEL and other AGEs	Microbial conjugation, reduction, degradation or fermentation changes may alter bioactivation, barrier injury and oxidative stress	Beer et al. (2019), Chen and Guo (2021), van Dongen et al. (2021)
Mycotoxins	Contaminated grains, maize, peanuts, nuts, spices, dried fruits and feed-derived animal products	AFB1, ochratoxin A, deoxynivalenol, zearalenone	Dysbiosis, altered bile acid signaling, LPS translocation and microbial metabolite changes may modify toxicity	Liu et al. (2023), Ye et al. (2023, 2024)
Pesticide and fungicide residues	Fruits, vegetables, grains, tea and contaminated agricultural products	Glyphosate, chlorpyrifos, pyrethroids, neonicotinoids, prothioconazole	May shift xenobiotic-degrading functions, aromatic amino acid metabolism, SCFAs, permeability and immune signaling	Meng et al. (2021), Li et al. (2025), Wei et al. (2025)
Food-contact chemicals	Canned foods, bottled beverages, packaged foods and food-contact materials	BPA, phthalates, styrene oligomers, plasticizers	Potential perturbation of bile acid, steroid and lipid metabolism, barrier function and microbial resilience	Lai et al. (2016), Priego et al. (2021)
Persistent and particulate contaminants	Seafood, drinking water, salt, packaged foods, food packaging and environmental entry into food chains	PFAS, MPs/NPs, PE, PP, PS, PET	Potential changes in microbial diversity, bile acids, lipid metabolism, SCFAs, oxidative stress and epithelial permeability	Hampson et al. (2024), Liang et al. (2024), Gao et al. (2025)
Potentially toxic elements	Rice, cereals, seafood, vegetables, drinking water and foods from polluted areas	Cadmium, lead, arsenic, mercury	May affect microbial richness, metal-resistance genes, SCFA production, bile acid metabolism and gut barrier integrity	He et al. (2020), Li X. et al. (2022), Li Y. et al. (2022)
Microbiota-transformable food bioactives	Fruits, vegetables, tea, coffee, cocoa, berries, nuts, spices and plant-based diets	Polyphenols including resveratrol, quercetin, catechins and curcumin	Deglycosylation, dehydroxylation, ring fission and phenolic-acid production may generate bioactive derivatives	Németh et al. (2003), Braune and Blaut (2016), Bouranis et al. (2021)

AFB1, aflatoxin B1; AGE, advanced glycation end product; AGEs, advanced glycation end products; BPA, bisphenol A; CEL, Nε-carboxyethyllysine; CMC, carboxymethylcellulose; CML, Nε-carboxymethyllysine; HAAs, heterocyclic aromatic amines; HCAs, heterocyclic amines; LPS, lipopolysaccharide; MPs, microplastics; NPs, nanoplastics; PAHs, polycyclic aromatic hydrocarbons; P80, polysorbate 80; PE, polyethylene; PET, polyethylene terephthalate; PFAS, per- and polyfluoroalkyl substances; PP, polypropylene; PS, polystyrene; SCFA, short-chain fatty acid; SCFAs, short-chain fatty acids.

The strength of evidence differs substantially within these additive classes. CMC and P80 have the clearest mechanistic support from animal and ex vivo human-community models showing direct effects on microbial function and inflammatory potential (Naimi et al., 2021; Chassaing et al., 2022). Evidence for lecithin, sucrose fatty acid esters and mono−/diglycerides is more limited, depends strongly on the experimental system and should not be generalized as a class effect (Sellem et al., 2023). For non-nutritive sweeteners, saccharin and sucralose have produced microbiota-dependent metabolic responses in selected human and experimental studies, whereas null or heterogeneous findings remain common (Zhang R. et al., 2023); compound identity, dose and baseline microbiota therefore need to be reported separately.

Food processing and high-temperature cooking generate another important group of dietary xenobiotics, including HCAs, PAHs, nitroso compounds and advanced glycation end-products (AGEs) (Phuong-Nguyen et al., 2023). These compounds are particularly relevant to food science and nutrition because their formation depends on processing conditions, food matrix and cooking temperature. The human fecal microbiota can metabolize foodborne HCAs through reuterin conjugation and related transformations, with substrate structure and culture conditions shaping the products formed (Nogacka et al., 2019). For AGEs, experimental studies indicate that high-AGE diets can remodel the gut microbiota, impair barrier function, increase inflammatory burden and raise AGE accumulation in plasma, liver and kidney. Some changes appear partially reversible after a low-AGE dietary intervention (Beer et al., 2019; Chen and Guo, 2021; van Dongen et al., 2021).

Processing parameters determine both the amount and the profile of generated chemicals. HCA formation generally increases with higher temperature and longer heating and depends on creatinine, amino acid and reducing-sugar precursors, whereas water-rich conditions can dilute reactants and change product spectra (Bordas et al., 2004). PAH formation is promoted by incomplete combustion, smoke deposition, direct flame contact and pyrolysis of dripping fat. AGE formation is favored by dry, high-temperature, low-moisture processing and is modified by pH, time and the relative abundance of reducing sugars, lipids and amino groups (Kim et al., 2021; Şen and Gökmen, 2022). Consequently, the same nominal food can deliver markedly different exposure according to cooking geometry, doneness and matrix composition.

Food-chain contaminants and emerging pollutants widen the exposure landscape beyond conventional additives. Persistent organic pollutants, pesticides, and fungicides can interact with the gut microbiota and may contribute to metabolic risk (Barra et al., 2025). MPs are a prominent emerging example because ingestion can place particles in direct contact with the intestinal ecosystem. Current evidence links MP exposure with altered microbial composition, metabolic dysfunction, mucosal inflammation and chronic disease-related phenotypes (Bora et al., 2024; Thin et al., 2025). For this Review, MPs are considered as food-chain exposures that may connect environmental contamination, intestinal ecology and host disease rather than as an environmental issue alone.

Potentially toxic elements and persistent PFAS require an exposure interpretation distinct from short-lived additives. Their persistence and transfer through feed, water, crops, livestock and aquatic food webs can produce bioaccumulation, with fish, seafood, meat, eggs, cereals and drinking water contributing differently across regions (Li Y. et al., 2022). Market-basket data also show that estimated PFAS intake varies by compound and population (Vestergren et al., 2012). Most experimental microbiome studies use doses and exposure windows that exceed habitual human intake; they are informative for mechanism but cannot be assumed to represent chronic low-dose dietary risk without internal-dose confirmation.

For MPs and nanoplastics (NPs), particle diameter, polymer identity, shape, surface oxidation or aging, adsorbed biomolecules and co-released plasticizers or stabilizers can alter dispersion, epithelial contact and microbial colonization. NPs may cross biological barriers more readily than larger particles, whereas larger particles can still modify mucus, tight junctions or luminal ecology (Shan et al., 2022). Simulated digestion further shows polymer-specific surface changes and microbiota interactions (Tamargo et al., 2022). These studies remain predominantly experimental and do not establish a uniform human disease effect.

Risk depends on more than the presence of a xenobiotic. Dose, food matrix, processing method, heating temperature and baseline microbiota can all modify biological response (Suez et al., 2022; Zapico et al., 2022). The same compound may therefore follow different host-specific trajectories, including detoxification, bioactivation, barrier injury or inflammatory amplification (Culp et al., 2024; Daniel et al., 2024). This is why dietary xenobiotic risk assessment should consider chemical exposure and microecological context together.

Food-matrix effects operate before and during microbial exposure. Dietary fat can increase micellar solubilization of lipophilic chemicals, whereas soluble or insoluble fiber can bind compounds, change transit, redistribute exposure toward the colon and alter the fermentative environment (Zhang L. et al., 2023; Li F. et al., 2026). Cell-wall structure, protein binding and processing-driven disruption of the matrix determine release kinetics (Edwards et al., 2021; Li et al., 2021). Exposure estimates based on an isolated chemical therefore cannot be transferred directly to a whole food without considering bioaccessibility, residence time and the microbial substrates supplied by the same meal.

## 3 Gut microbial handling of food-derived xenobiotics

### 3.1 Biotransformation principles and enzyme repertoire

After ingestion, the gut microbiota is often the first biological system to encounter food-derived xenobiotics. Gut microorganisms can alter dietary compounds, environmental toxicants and drugs, thereby changing absorption, exposure level, toxicity and clearance (Collins and Patterson, 2020). These reactions can detoxify parent compounds, facilitate excretion or generate more reactive metabolites. Hydrolysis and related transformations can also produce smaller intermediates that enter downstream host or microbial pathways (Németh et al., 2003; Bouranis et al., 2021). The microbiota should therefore be viewed as a determinant of xenobiotic outcome, not simply as a parallel metabolic route.

Three evidence levels are distinguished throughout this Review. Direct microbial biotransformation requires evidence of parent-compound depletion, product formation, isotope tracing, or an identified strain, gene or enzyme. Ecological perturbation refers to changes in community composition, abundance or function after exposure and does not by itself demonstrate metabolism of the parent compound. Downstream host mediation refers to barrier, immune, receptor or organ effects that are altered by microbiota manipulation. These levels can be linked experimentally, but a compositional association alone is insufficient to establish either biotransformation or microbiota-mediated organ toxicity.

A phase I/phase II analogy can be useful only as a descriptive shorthand. Microbial functionalization includes reduction, hydrolysis, dealkylation or related reactions, whereas deconjugation and reconjugation alter polarity and enterohepatic cycling; however, microbial pathways are not organized like host hepatic phases and often reverse host conjugation (Lucas et al., 2021; Culp et al., 2024). Reaction opportunity also varies spatially: the largely anaerobic colon favors reductive chemistry, while more oxygenated mucosal niches support different redox activities (Penumutchu et al., 2023; Zong et al., 2024). Longer transit can increase substrate-enzyme contact, but also changes substrate depletion, community growth and absorption, so residence time is a modifier rather than a simple dose multiplier.

The enzymatic basis for this role is now clearer. Das et al. analysed 397 gut metagenomes from individuals of different ages and eight countries or regions, identifying substantial variation in xenobiotic-metabolizing enzyme abundance across genera. Genera could be stratified into low, moderate and high metabolic-capacity tiers, with several phase I and phase II enzyme classes linked to this stratification (Das et al., 2016). Zimmermann et al. (2019) then screened 76 human gut bacterial strains against 271 orally administered drugs and showed that many metabolic phenotypes could be traced to specific strains, genes and enzymes. Table 2 summarizes representative microbial enzymes, substrates, transformation outcomes and disease links. Together, these studies show that xenobiotic metabolism is governed by identifiable microbial functions rather than by community composition alone.

**Table 2:** Microbial functions involved in xenobiotic biotransformation.

Microbial function/enzyme	Representative microbial carriers	Typical substrates	Transformation	Functional consequence	References
Glycosidases	*Bacteroides*, *Bifidobacterium*, *Lactobacillus*, *Eubacterium*, *Blautia*	Polyphenol glycosides, isoflavones, lignans and plant small molecules	Deglycosylation and downstream metabolite generation	Often increases bioavailability or produces bioactive metabolites relevant to barrier and metabolic signaling	Németh et al. (2003), Braune and Blaut (2016), Bouranis et al. (2021)
GUS	*Bacteroides*, *Clostridium*, *Escherichia* and other *Firmicutes*/*Bacteroidetes*/*Proteobacteria* members	Glucuronidated xenobiotics, phenolics, hormones and drugs	Deglucuronidation and reactivation after host phase II conjugation	Can regenerate active or toxic aglycones and prolong enterohepatic cycling	Brossier et al. (2024)
Sulfatases	*Bacteroides*, *Clostridium* and other anaerobic commensals	Sulfated phenolics, hormones and bile acid derivatives	Desulfation and altered enterohepatic cycling	Context-dependent detoxification or bioactivation; may change epithelial exposure	Ervin et al. (2020)
BSH	*Lactobacillus*, *Bifidobacterium*, *Bacteroides*, *Clostridium*, *Enterococcus*	Glycine- or taurine-conjugated bile acids	Bile acid deconjugation	Alters FXR/TGR5/PXR signaling, bile acid pools and microbial ecology	Begley et al. (2006)
Hydroxysteroid dehydrogenases/7α-dehydroxylases	*Clostridium scindens*, *C. hylemonae*, *C. hiranonis*, *Eggerthella lenta* and related anaerobes	Primary and secondary bile acids	Oxidation, epimerization and 7α-dehydroxylation	Generates secondary bile acids with altered receptor activity; may protect or injure depending on context	Funabashi et al. (2020), Ridlon et al. (2020)
Azoreductases	*Escherichia coli*, *Enterococcus*, *Clostridium*, *Eubacterium* and *Bacteroides species*	Azo food dyes and azo-containing xenobiotics	Reductive cleavage of azo bonds	May generate aromatic amines; effect depends on substrate, exposure and microbial context	Rafii et al. (1997), Zahran et al. (2019), Elder et al. (2023)
Nitroreductases	*Bacteroides*, *Escherichia coli*, *Clostridium*, *Enterobacteriaceae* and other anaerobes	Nitroaromatic and nitroheterocyclic compounds, including nitro-PAHs	Reduction to hydroxylamines or amines	Can bioactivate mutagenic intermediates or detoxify depending on oxygen and substrate	Garcia et al. (2022)
Tryptophanase/TnaA/TIL	*Escherichia*, *Proteus*, *Bacteroides*, *Clostridium*, *Peptostreptococcus*	Dietary tryptophan	Indole formation and downstream host conversion to indole derivatives or indoxyl sulfate	Indoles may support barrier/AhR signaling, whereas indoxyl sulfate is a uremic toxin	Venkatesh et al. (2014), Dodd et al. (2017), Graboski et al. (2023)
TMA-forming enzymes (CutC/D, CntA/B)	*Firmicutes*, *Actinobacteria*, *Proteobacteria*, *Anaerococcus*, *Clostridium*, *Escherichia*, *Acinetobacter*	Choline, L-carnitine, γ-butyrobetaine and betaine	Formation of TMA followed by hepatic oxidation to TMAO	Links diet, microbial metabolism and cardiometabolic/cardiorenal risk	Romano et al. (2015), Rath et al. (2017)

AhR, aryl hydrocarbon receptor; BSH, bile salt hydrolase; CntA/B, carnitine oxygenase/reductase; CutC/D, choline trimethylamine-lyase system; FXR, farnesoid X receptor; GUS, β-glucuronidase; PAHs, polycyclic aromatic hydrocarbons; PXR, pregnane X receptor; TGR5, Takeda G protein-coupled receptor 5; TIL, tryptophan indole-lyase; TMA, trimethylamine; TMAO, trimethylamine-N-oxide; TnaA, tryptophanase.

### 3.2 Metagenomic/metabolomic signatures and inter-individual variability

Recent work has shifted the field from asking whether the microbiota metabolizes xenobiotics to asking which organisms, genes and products determine the outcome (Zimmermann et al., 2019). Culp et al. mapped dietary xenobiotic-microbiota interactions and showed that gut communities can detoxify some compounds while toxifying others, with substantial inter-individual variation (Culp et al., 2024). The same study localized genes and enzymes involved in resveratrol detoxification. This illustrates why metagenomics, metabolomics and functional experiments need to be integrated: taxonomy alone cannot identify the metabolites produced or the host risk created.

Inter-individual variability is a central feature of microbial xenobiotic metabolism. Das et al. found geographic and interpersonal differences in xenobiotic-metabolizing enzyme abundance (Das et al., 2016). Culp et al. further showed that variation in dietary xenobiotic transformation can reflect the presence or absence of specific commensals and their metabolic capacities (Culp et al., 2024). As a result, the same food exposure may be detoxified in one host but bioactivated or amplified in another (Suez et al., 2022). Gut-liver-kidney risk assessment therefore needs to account for host microecology as well as exposure level.

Microbial xenobiotic capacity also changes across the life course and populations. Metagenomic analyses report age- and geography-associated differences, while diet, medication, environmental exposure and host genetics can select different functional repertoires (Das et al., 2016). The degree to which a person’s transformation capacity is stable over months or years remains poorly defined and may differ by pathway. Risk assessment should therefore represent uncertainty and susceptible tails of the response distribution. At present, evidence is insufficient to define regulatory ‘microbiome-protected’ or ‘microbiome-vulnerable’ groups or to revise acceptable daily intakes directly; prospective exposure-response studies are required first.

### 3.3 Ecological remodeling and functional perturbation by xenobiotic exposure

Food-derived xenobiotics are not only microbial substrates. They can also reshape the gut ecosystem. Food additives have been associated with altered microbial composition, weakened intestinal homeostasis and stronger inflammatory responses (Li P. et al., 2024; Gao et al., 2025). Colonic fermentation models have tested microbiota from healthy donors and patients with inflammatory bowel disease (IBD). In these systems, P80, sucralose, titanium dioxide, sodium nitrite and maltodextrin changed microbial composition and metabolic activity. IBD-derived microbiota showed greater sensitivity (Gonza et al., 2024). These additives promoted Enterococcus, Escherichia and Streptococcus and reduced *Akkermansia muciniphila* in IBD donor microbiota. P80 also reduced Roseburia and *Faecalibacterium prausnitzii*, suggesting preferential loss of butyrate-producing bacteria alongside enrichment of potentially pro-inflammatory taxa (Loayza et al., 2023; Gonza et al., 2024).

The functional consequences extend beyond taxonomic change. *In vitro* work indicates that multiple food additives can modify microbial metabolite production and enrich signatures associated with intestinal inflammation and fibrosis (Gonza et al., 2024; Li X. et al., 2024). Selected additives can also weaken tight junction function, thin the mucus layer and disrupt barrier homeostasis, often alongside reduced microbial metabolic activity (Li P. et al., 2024). Thus, additive effects should be interpreted across several linked layers: microbial taxa, short-chain fatty acid (SCFA) production, aryl hydrocarbon receptor (AhR) activity and barrier-related metabolic networks.

Xenobiotic exposure can also act as selective pressure within the gut ecosystem (Chassaing et al., 2015). *In vitro* models show that food additives may expand taxa associated with inflammation and fibrosis while suppressing health-associated genera (Gonza et al., 2024). This selection does not require total community collapse (Chassaing et al., 2017). It may arise through changes in substrate availability, mucus-layer conditions, redox state or host immune pressure, allowing tolerant taxa to gain an advantage (Desai et al., 2016; Byndloss et al., 2017). Such ecological selection helps explain why the same additive can produce different degrees of perturbation across hosts.

Ecological remodeling can arise through direct and indirect routes. Some chemicals inhibit growth, damage membranes or alter nutrient use directly; others first change host mucus secretion, luminal pH, oxygen leakage, immune pressure or bile acid composition (Nayak et al., 2021; Paone et al., 2024). Loss of a keystone function can then redirect cross-feeding and competitive networks even when total diversity changes little (Marcelino et al., 2023; Weiss et al., 2023). Conversely, taxonomic replacement may preserve a function through redundancy, and an apparently recovered community may retain altered metabolic output (Jiang et al., 2025). Both resistance and functional resilience should therefore be measured rather than inferred from taxonomy alone.

Non-nutritive sweeteners provide a useful example of host-specific response. Clinical trials have reported heterogeneous effects of sucralose, saccharin and aspartame on gut microbial composition, with some studies showing changes and others showing little effect (Conz et al., 2023). A randomized controlled trial in 2022 showed that sweetener effects on glucose tolerance were individualized and microbiota-dependent (Suez et al., 2022). This evidence supports a restrained interpretation: sweeteners do not produce one uniform microbial effect, and baseline host microecology may strongly modify metabolic response (Gauthier et al., 2024).

MPs extend the review from conventional additives to emerging food-chain contaminants. Ingestion can expose the intestinal ecosystem to MPs and NPs, and experimental studies suggest effects on oxidative stress, inflammation, barrier integrity and particle-related toxicity (Schwabl et al., 2019; Liang et al., 2021). Human and experimental evidence also links MP exposure with microbial shifts and metabolic dysfunction (Thin et al., 2025). These findings broaden the food-derived xenobiotic framework, but they remain less mature than the evidence for selected sweeteners and emulsifiers (Gao et al., 2025).

Overall, food-derived xenobiotics affect the gut microbiota in three recurring ways. They can change microbial composition and diversity, alter metabolic activity and ecological competition, and produce host-specific effects shaped by baseline microbiota and disease background. For chronic disease along the gut-liver-kidney axis, xenobiotic exposure is therefore best interpreted as a chemical pressure filtered through microbial ecology.

## 4 Cross-organ mechanisms linking microbial xenobiotic metabolism to host physiology

### 4.1 Gut barrier disruption and immune-metabolic inflammation

The intestinal mucosal barrier, composed of epithelial cells, tight junctions, mucus and mucosal immunity, limits entry of luminal microbial products and xenobiotic chemicals into host circulation (Johansson et al., 2008; Vaishnava et al., 2011). Microbial metabolites help regulate this barrier. SCFAs are classic barrier-supporting metabolites, whereas food- and microbe-derived tryptophan metabolites can activate AhR and influence barrier and immune homeostasis (Venkatesh et al., 2014; Ghosh et al., 2021). When xenobiotics disturb these metabolic networks, the first detectable consequence may be barrier dysfunction rather than overt organ injury (Scott et al., 2020).

Barrier integrity depends on more than epithelial permeability. Goblet cells continuously renew the mucin 2-rich mucus layer, whose organization differs between small intestine and colon and controls how closely microbes and particles approach the epithelium (Nyström et al., 2021; Gustafsson and Hansson, 2025). Occludin, claudins and zonula occludens-1 coordinate tight-junction selectivity through kinase, cytoskeletal and inflammatory pathways (Oami et al., 2024; Zhu et al., 2024). Regional physiology also matters: the duodenum and jejunum favor rapid absorption, the ileum couples bile acid uptake to farnesoid X receptor (FXR) signaling, and the colon combines prolonged residence with dense microbial fermentation (Liu et al., 2022; Zwick et al., 2024). A xenobiotic can therefore have different access, transformation and barrier effects along the gastrointestinal tract.

SCFAs are not interchangeable biomarkers. Acetate is the most abundant and circulates widely; propionate is substantially extracted by the liver; and butyrate is a preferred oxidative fuel for colonocytes and can also inhibit histone deacetylases and signal through G-protein-coupled receptors (Kibbie et al., 2021; Kirschner et al., 2021). Butyrate signaling through G protein-coupled receptor 109A supports epithelial and immune homeostasis, whereas butyrate oxidation sustains colonocyte energy metabolism (Donohoe et al., 2011; Singh et al., 2014). Millimolar luminal concentrations contrast with much lower systemic concentrations, so fecal abundance does not directly indicate mucosal flux or exposure in liver and kidney.

Barrier impairment allows microbial molecules such as lipopolysaccharide (LPS) to cross the epithelium and enter circulation. Persistent low-grade endotoxin exposure can drive inflammation, oxidative stress and metabolic dysfunction, and has been linked to obesity, impaired glucose metabolism and cardiovascular risk (Piya et al., 2013). Thus, food-derived xenobiotics may affect distant organs indirectly by first changing microbial ecology, metabolite production and barrier permeability.

### 4.2 Microbial metabolites and host receptor signaling

Bile acids connect food intake, microbial metabolism and host signaling. FXR and Takeda G protein-coupled receptor 5 (TGR5) are highly expressed in the intestinal epithelium and contribute to bile acid homeostasis, barrier function and immune balance (Song et al., 2025). Secondary bile acid transformation by gut microbes can further modify these receptor pathways. Pregnane X receptor (PXR) is another relevant nuclear receptor because xenobiotics, dietary factors and microbe-derived metabolites can regulate its activity in bile acid homeostasis and xenobiotic biotransformation (Dutta et al., 2022). These receptor networks help explain how microbial metabolism can influence both intestinal integrity and hepatic inflammatory status.

Most bile acids are reclaimed in the terminal ileum through the apical sodium-dependent bile acid transporter and exported basolaterally through organic solute transporter alpha and beta subunits before hepatic uptake and resecretion (Wang et al., 2026). Microbial deconjugation and secondary transformation change the composition and signaling potency of the returning pool. Intestinal FXR induces fibroblast growth factor 15 (FGF15) in mice and its human ortholog fibroblast growth factor 19 (FGF19), which signal to hepatic fibroblast growth factor receptor 4 to restrain bile acid synthesis (Xiao et al., 2025; Fruzyna et al., 2026). Xenobiotic effects may also arise through altered intestinal or hepatic transporter expression, but direct transporter evidence remains compound-specific (Degirolamo et al., 2014).

AhR, PXR and constitutive androstane receptor (CAR) were once viewed mainly as xenobiotic sensors, but they also regulate intestinal homeostasis. AhR responds to environmental and diet-related ligands as well as microbe-derived tryptophan metabolites, supporting barrier protection and local immune balance (Vázquez-Gómez et al., 2023). PXR can be activated by microbe-derived indole-3-propionic acid and secondary bile acids, and can influence barrier integrity, inflammation and gut-liver metabolic networks (Staudinger et al., 2001; Venkatesh et al., 2014). PXR and CAR may therefore act as shared hubs between xenobiotic exposure, host-microbiota interaction and chronic disease phenotypes (Feng et al., 2025).

These receptors have a dual identity. As xenobiotic sensors, AhR, PXR and CAR induce enzymes and transporters that limit or redistribute foreign-chemical exposure (Robbins et al., 2022). As physiological regulators, the same receptors respond to endogenous or microbial ligands and coordinate bile acid, lipid, glucose, immune and barrier homeostasis (Flannigan et al., 2023; Willemsen et al., 2024). Xenobiotic perturbation can therefore reflect excessive, deficient or mistimed activation of a normal homeostatic circuit rather than a separate toxicant-only pathway.

Taken together, food-derived xenobiotics may influence chronic disease by more than direct chemical toxicity. They can alter microbial structure and function, disturb SCFA, bile acid and tryptophan metabolism, and promote barrier disruption, endotoxemia, immune imbalance and metabolic reprogramming. This mechanism map supports an integrated discussion of gut-liver-kidney outcomes and candidate microbiome-targeted interventions.

## 5 Gut-liver-kidney outcomes of selected food-derived xenobiotics

### 5.1 Hepatic first-pass exposure, steatotic liver disease mechanisms and aflatoxin B1 injury

After oral intake, food-derived xenobiotics are absorbed, transformed or selected in the intestine before reaching the liver through the portal vein (Culp et al., 2024; Muñoz et al., 2025). The liver is therefore both an exposure organ and a target for toxicity amplification (Figure 2). Aflatoxin B1 (AFB1), a foodborne mycotoxin, is a representative model. In experimental studies, AFB1 induces liver injury through oxidative stress, mitochondrial dysfunction, inflammation and gut microbial dysbiosis (Xu et al., 2021; Cheng et al., 2025). Gut microbiota dysbiosis is closely associated with AFB1 hepatotoxicity (Moloi et al., 2024).

**Figure 2:**
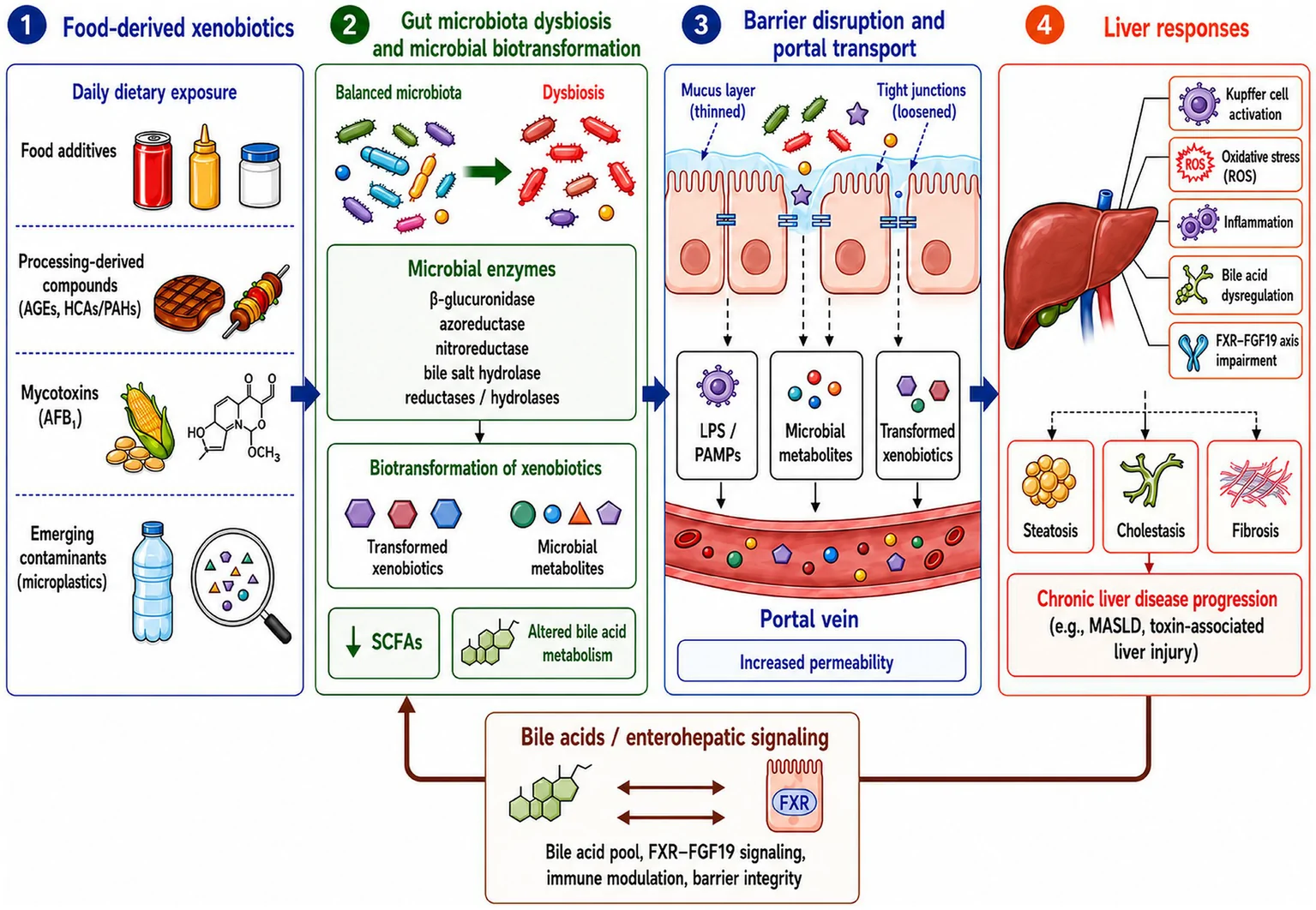
Gut–liver axis mechanisms linking microbial xenobiotic biotransformation to chronic liver disease. Food-derived xenobiotics, including food additives, processing-derived compounds, mycotoxins, and emerging contaminants, enter the gastrointestinal tract through daily dietary exposure. These compounds can reshape gut microbial composition, promote dysbiosis, and undergo microbial biotransformation mediated by enzymes such as β-glucuronidase, azoreductase, nitroreductase, bile salt hydrolase, and other reductases or hydrolases. Microbial biotransformation generates transformed xenobiotics and microbial metabolites, accompanied by decreased short-chain fatty acids and altered bile acid metabolism. Dysbiosis and barrier impairment, characterized by mucus layer thinning and loosened tight junctions, increase intestinal permeability and facilitate the translocation of lipopolysaccharide, pathogen-associated molecular patterns, microbial metabolites, and transformed xenobiotics into the portal circulation. These gut-derived signals reach the liver through the portal vein and trigger Kupffer cell activation, oxidative stress, inflammatory responses, bile acid dysregulation, and impairment of the FXR–FGF19 axis. Together, these processes contribute to steatosis, cholestasis, fibrosis, and chronic liver disease progression, including MASLD and toxin-associated liver injury. Bile acids and enterohepatic signaling further mediate bidirectional communication between the gut microbiota and liver by regulating the bile acid pool, FXR–FGF19 signaling, immune modulation, and barrier integrity. Abbreviations: AFB1, aflatoxin B1; AGEs, advanced glycation end products; BSH, bile salt hydrolase; FGF19, fibroblast growth factor 19; FXR, farnesoid X receptor; HCAs, heterocyclic amines; LPS, lipopolysaccharide; MASLD, metabolic dysfunction-associated steatotic liver disease; PAHs, polycyclic aromatic hydrocarbons; PAMPs, pathogen-associated molecular patterns; ROS, reactive oxygen species; SCFAs, short-chain fatty acids.

AFB1 evidence must be interpreted by exposure regime. Acute or short-term high-dose animal models are valuable for identifying oxidative, pyroptotic and microbiota-dependent mechanisms, but they do not reproduce the chronic low-dose intake that characterizes many human settings (Wakabayashi et al., 2026). Contamination varies with crop, geography, climate, fungal growth and post-harvest moisture and temperature; poor storage can increase exposure long after harvest. Human inference therefore requires measured food contamination or internal biomarkers rather than nominal animal dose alone.

MASLD illustrates how microbiota-related mechanisms intersect with a common chronic liver phenotype. Its global burden is increasing, but this burden cannot be attributed to individual dietary xenobiotics (Huang et al., 2025). Western dietary patterns characterized by high energy density, high saturated fat or free sugars and low fiber can independently reshape microbial ecology, barrier function and hepatic lipid handling (Yang et al., 2023). Accordingly, associations between a specific exposure, dysbiosis and MASLD remain vulnerable to confounding by the overall dietary pattern, obesity, medication and metabolic disease. The revised interpretation treats xenobiotics as potential modifiers within a multifactorial disease process rather than as established population-level causes.

Bile acid-FXR signaling is a key route through which gut microbial metabolism may influence hepatic response. Changes in bile acid composition are associated with metabolic disease, and FXR helps regulate bile acid homeostasis, lipid metabolism and intestinal integrity (Li Y. et al., 2024; Tang et al., 2024). This pathway is particularly relevant in AFB1 models. Experimental studies show that AFB1-induced liver injury can involve gut microbiota-bile acid-FXR disruption, with effects on inflammation, pyroptosis and hepatic metabolism (Ye et al., 2023).

Evidence from toxicant-induced liver injury is used here as a comparator, not as proof for all dietary xenobiotics. Food-relevant experimental evidence comes mainly from AFB1, pesticide, fungicide and microplastic models. In these systems, gut microbial dysbiosis, barrier injury, bile acid-FXR disruption, oxidative stress, inflammation and pyroptosis have been linked to hepatic injury (Meng et al., 2021; Liu et al., 2023; Xu et al., 2024; Li et al., 2025; Wei et al., 2025). The comparator evidence should therefore be treated as mechanism-generating support for gut-liver crosstalk.

Original studies clarify this chain. In mice, AFB1 altered gut microbial composition, damaged the colonic barrier and promoted hepatocyte pyroptosis and inflammatory responses. Antibiotic depletion weakened these intestinal and hepatic effects, supporting a microbiota-dependent component of AFB1 injury (Ye et al., 2023). Other studies link AFB1 with gut microbial-metabolite disruption and liver pyroptosis (Ye et al., 2024). These findings make AFB1 a useful foodborne model for studying how microbial ecology can amplify hepatic toxicity.

Overall, the gut-liver axis organizes mechanistic evidence without implying a universal causal pathway. Microbial transformation or ecological disruption may modify lipid regulation, inflammation, pyroptosis and fibrosis through bile acids, intestinal FXR-FGF15 signaling in mice or FXR-FGF19 signaling in humans, LPS, SCFAs and other metabolites. AFB1 provides a food-derived mechanistic model, whereas MASLD and non-food toxicant models provide contextual rather than direct proof. The predominance of animal and *in vitro* evidence requires cautious translation to habitual human exposure (Table 3).

**Table 3:** Representative food-chain xenobiotics with gut-mediated liver or kidney functional outcomes.

Exposure	Food-chain/source relevance	Evidence model	Microbial/metabolic finding	Functional outcome	Evidence strength	References
Dietary AGEs	Baked, fried and roasted high-AGE foods	Mouse high-AGE diet models; reversibility assessment	Altered microbial composition, reduced anti-inflammatory tone and increased AGE burden	Systemic inflammation and liver/kidney AGE accumulation	Animal dietary model with food-processing relevance	Chen and Guo (2021), van Dongen et al. (2021)
AFB1	Contaminated grains, peanuts, nuts and feed-derived foods	Mouse models; antibiotics, FMT and FXR-related intervention	Dysbiosis, altered bile acid-FXR/FGF15 signaling, LPS translocation, pipecolic acid/norepinephrine changes	Inflammatory liver injury, pyroptosis, oxidative stress and bile acid dysregulation	Animal mechanistic; causal support from microbiota manipulation	Liu et al. (2023), Ye et al. (2023, 2024)
Fungicide residues	Agricultural products and food-chain residues	Mouse gut microbiota, barrier and liver translocation analyses	Gut dysbiosis, cecal metabolic perturbation and impaired barrier function	Liver inflammation and injury with bacterial translocation	Animal mechanistic	Meng et al. (2021)
Organophosphate pesticide	Fruit, vegetable and grain residues	Dose–response C57BL/6 J mouse model	Dose-dependent dysbiosis and hepatic metabolic perturbation	Liver injury associated with metabolic imbalance	Animal dose–response	Wei et al. (2025)
Glyphosate	Food-chain herbicide residue exposure	Mouse 16S rDNA sequencing and serum metabolomics	Altered genera and serum metabolites; pentose phosphate and pyrimidine pathway changes	Hepatic edema, inflammatory infiltration and biochemical injury markers	Animal multi-omics association	Li et al. (2025)
BPA	Food-contact materials, canned foods and packaged foods	Dietary/chronic BPA mouse models	Reduced microbial diversity, *Proteobacteria* enrichment and functional disruption	Tubular injury, renal inflammation and fibrosis markers	Animal + observational plausibility	Lai et al. (2016), Priego et al. (2021)
PS-MPs	Packaging, water and food-chain particulate contamination	Mouse exposure model	Barrier dysfunction, LPS translocation and C5a/C5aR activation	CKD-like injury, glomerular sclerosis and interstitial fibrosis markers	Animal mechanistic/emerging	Liang et al. (2024)
Cadmium	Rice, cereals, vegetables, seafood and polluted food chains	Chronic oral mouse exposure; dietary fiber intervention	Reduced microbial richness and SCFA-producing bacteria; altered absorption/accumulation	Kidney injury with creatinine/urea changes	Animal + dietary intervention	He et al. (2020), Li X. et al. (2022), Li Y. et al. (2022)
)PE/PET MPs	Seafood, water, salt, packaging and environmental food-chain entry	Mouse oral/chronic exposure models	Altered microbial structure, inflammatory pathway activation and hepatic lipid disruption	Liver injury or lipid accumulation in experimental models	Animal mechanistic/emerging	Xu et al. (2024), Lu et al. (2025)
Melamine	Food adulteration or contamination scenarios	Rat model and *in vitro* fecal bacterial cultures	Microbial conversion of melamine to cyanuric acid	Melamine-cyanurate crystal formation and renal toxicity	Animal + mechanistic in vitro; strong biotransformation example	Zheng et al. (2013)
Ochratoxin A (OTA)	Contaminated cereals, coffee, dried fruits, spices and feed-derived foods	Mouse oral exposure; inulin/postbiotic intervention studies	OTA-induced dysbiosis, altered SCFAs and inflammatory/pyroptosis signaling	Renal inflammation and tubular injury	Animal + intervention	Jiang et al. (2024), Wang J. et al. (2024)

AFB1, aflatoxin B1; AGE, advanced glycation end product; AGEs, advanced glycation end products; BPA, bisphenol A; C5a, complement component 5a; C5aR, complement component 5a receptor; CKD, chronic kidney disease; FGF15, fibroblast growth factor 15; FMT, fecal microbiota transplantation; FXR, farnesoid X receptor; IL-10, interleukin-10; LPS, lipopolysaccharide; MPs, microplastics; NF-κB, nuclear factor kappa B; NLRP3, NOD-like receptor family pyrin domain-containing 3; OTA, ochratoxin A; PE, polyethylene; PET, polyethylene terephthalate; PS-MPs, polystyrene microplastics; rDNA, ribosomal DNA; SCFAs, short-chain fatty acids; TLR2, Toll-like receptor 2; TLR4, Toll-like receptor 4.

### 5.2 Kidney outcomes, uremic toxin burden and microbiota-mediated injury

Kidney evidence comprises two distinct questions. First, selected food-chain xenobiotics, including melamine, ochratoxin A (OTA), cadmium, bisphenol A (BPA) and MPs, can produce renal injury in experimental systems, but direct microbial biotransformation has been demonstrated clearly only for selected examples such as melamine (Liang et al., 2024; Shi et al., 2025). Second, established CKD changes the intestinal environment, microbial metabolism and systemic clearance. These processes support a bidirectional gut-kidney axis but should not be used as proof that food-derived xenobiotics initiated CKD (Figure 3). The revised section therefore separates xenobiotic-induced renal injury from altered handling of microbial-host co-metabolites after renal function has declined.

**Figure 3:**
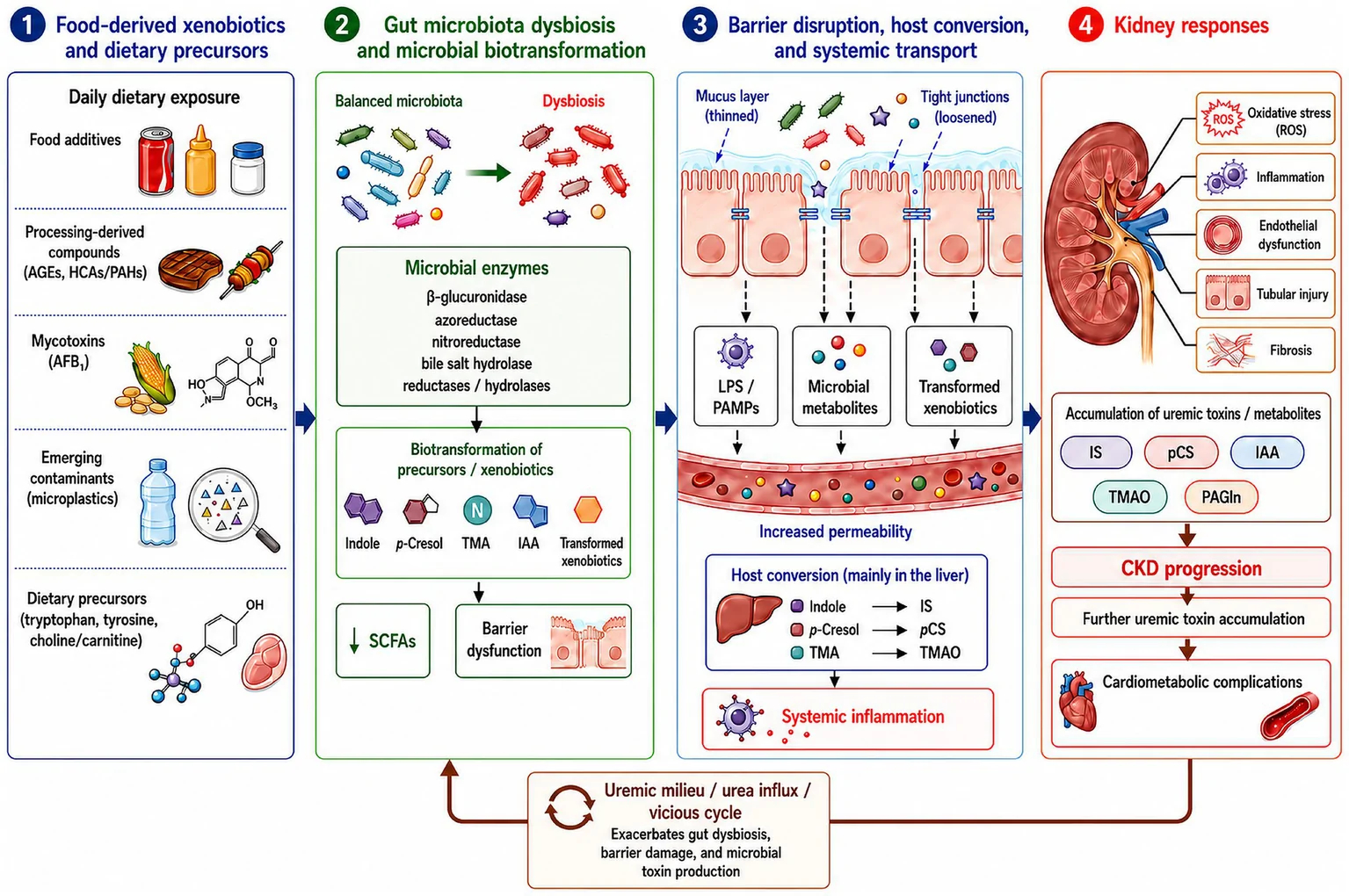
Gut–kidney axis mechanisms linking microbial xenobiotic biotransformation to chronic kidney disease. Food-derived xenobiotics and dietary precursors, including food additives, processing-derived compounds, mycotoxins, microplastics, tryptophan, tyrosine, and choline/carnitine, enter the gastrointestinal tract through daily dietary exposure. These compounds and precursors can reshape gut microbial composition, promote dysbiosis, and undergo microbial enzyme-mediated biotransformation through β-glucuronidase, azoreductase, nitroreductase, bile salt hydrolase, reductases, and hydrolases. This process generates microbial metabolites and transformed xenobiotics, including indole, p-cresol, trimethylamine, indole-3-acetic acid, and other xenobiotic-derived products, while reducing short-chain fatty acid production and impairing the intestinal barrier. Dysbiosis and barrier disruption increase intestinal permeability, facilitating the systemic translocation of lipopolysaccharide, pathogen-associated molecular patterns, microbial metabolites, and transformed xenobiotics. Some gut-derived metabolites are further converted by the host, mainly in the liver, into circulating uremic toxins, including indoxyl sulfate, p-cresyl sulfate, and trimethylamine-N-oxide. These circulating metabolites, together with inflammatory signals, contribute to oxidative stress, inflammation, endothelial dysfunction, tubular injury, and renal fibrosis. The accumulation of indoxyl sulfate, p-cresyl sulfate, indole-3-acetic acid, trimethylamine-N-oxide, and phenylacetylglutamine promotes chronic kidney disease progression and cardiometabolic complications. Conversely, the uremic milieu and increased urea influx further exacerbate gut dysbiosis, barrier injury, and microbial toxin production, forming a vicious gut–kidney axis cycle. AFB1, aflatoxin B1; AGEs, advanced glycation end products; BSH, bile salt hydrolase; CKD, chronic kidney disease; HCAs, heterocyclic amines; IAA, indole-3-acetic acid; IS, indoxyl sulfate; LPS, lipopolysaccharide; PAGln, phenylacetylglutamine; PAHs, polycyclic aromatic hydrocarbons; PAMPs, pathogen-associated molecular patterns; PBUTs, protein-bound uremic toxins; pCS, p-cresyl sulfate; ROS, reactive oxygen species; SCFAs, short-chain fatty acids; TMA, trimethylamine; TMAO, trimethylamine-N-oxide.

Protein-bound uremic toxins (PBUTs) are not dietary exogenous toxins. They are microbial-host co-metabolites whose dietary precursors enter distinct pathways. Tryptophan can be converted by gut bacteria to indole and then sulfated by the host to indoxyl sulfate (IS), whereas tyrosine and phenylalanine fermentation produces p-cresol that is converted mainly to p-cresyl sulfate (pCS) (Lin et al., 2025; Jiang et al., 2026). Production depends on substrate delivery and microbial enzymes, while circulating concentrations depend strongly on hepatic conjugation, tubular secretion, protein binding and renal clearance (Rong and Kiang, 2021). IS and pCS differ in precursor ecology and biological associations and should not be treated as one exposure. Prospective cohorts associate higher concentrations with adverse outcomes, but residual confounding by kidney function and inconsistent toxin-lowering intervention effects mean that causal contribution remains plausible rather than established (Wu et al., 2011; Chen et al., 2022a).

Declining kidney function can also change the intestinal habitat. Urea and other wastes accumulate in the gut lumen and select microbial communities that produce more uremic toxins, a pattern often described as uremic dysbiosis (Amini Khiabani et al., 2023). CKD is associated with altered gut microbial composition and function, which can promote toxin accumulation and disease progression (Rysz et al., 2021). This bidirectionality is important because dietary interventions may act through microbial metabolism as well as through nutrient restriction (Tsuji et al., 2024).

Gut-derived uremic toxins can damage more than the kidney. PBUTs contribute to cardiovascular and renal complications through inflammation, oxidative stress and endothelial dysfunction (Yu et al., 2011; Rossi et al., 2014). IS can also injure the intestinal barrier and promote immune imbalance, creating a loop between toxin accumulation, barrier dysfunction and systemic inflammation (Lau et al., 2018). This loop helps explain why renal impairment can intensify the biological consequences of the same dietary or microbial precursor exposure.

Intervention evidence is promising but not yet definitive. Meta-analyses of randomized trials suggest that microbiome-based strategies can reduce circulating IS and pCS in CKD (Chen et al., 2022a). Probiotics, synbiotics, prebiotics and related approaches may act by lowering precursor generation, improving barrier function or reshaping microbial structure (Yang C. -Y. et al., 2021). Dietary strategies, especially higher fiber approaches, may also reduce gut-derived toxin burden (Melekoglu and Samur, 2021). These findings support a nutritional logic for the gut-kidney axis, but effects vary across studies and outcomes (Luo et al., 2023; Liu et al., 2024).

Overall, the gut-kidney axis links diet, microbial metabolism and renal clearance through several non-equivalent routes. Dietary precursors can feed microbial pathways that generate host-conjugated solutes; selected xenobiotics can cause renal injury in experimental models; and CKD can independently intensify dysbiosis, barrier dysfunction and solute accumulation. Because reduced clearance can generate the same metabolite signature attributed to increased production, longitudinal measurements of intake, microbial flux and kidney function are required to determine directionality. Microbiota modulation may lower selected solutes, but evidence for slowing CKD progression or preventing food-xenobiotic injury remains insufficient.

### 5.3 Shared liver-kidney mechanisms and multi-omics readouts

The gut-liver-kidney axis should not be read as a linear connection among three organs. It is a network shaped by dysbiosis, barrier disruption, metabolic disturbance and immune amplification. Increased intestinal permeability can allow microbial metabolites and pathogen-associated molecular patterns (PAMPs) to reach the liver and kidney through portal or systemic circulation, where they may induce metabolic reprogramming, inflammation and oxidative stress (Anand and Mande, 2022; Fei et al., 2025). At the same time, hepatic and renal impairment can alter metabolite clearance and reshape the gut environment (Alobaidi, 2025).

Along this axis, the key signals are not a single molecule but multiple classes of microbe-derived metabolites and inflammatory mediators. Gut-derived metabolites can enter the liver through the portal vein, and activated immune cells can contribute to hepatic inflammatory responses through the lymphatic system (Eksteen et al., 2004; Orabi et al., 2021). The liver can then reshape gut microbial structure and function through bile acids and other metabolites. In the kidney, reduced renal function allows gut-derived PBUTs such as IS and pCS to accumulate. These toxins can impair the intestinal barrier, disrupt systemic homeostasis and aggravate inflammation and oxidative stress (Lin et al., 2011; Huang et al., 2020). Bilirubin biotransformation to urobilin by the gut microbiota has also been proposed as a measurable signal in the gut-liver-kidney axis (Kipp et al., 2025). This indicates that the axis includes both injury signals and potentially useful metabolic readouts.

Shared pathways also include one-carbon metabolism, nitrogen and urea handling, mitochondrial redox balance and systemic inflammatory signaling. The same metabolite may have different effects according to portal versus systemic concentration, receptor expression, hepatic transformation and renal clearance (Muñoz et al., 2025). Inflammation is a unifying amplifier across gut, liver and kidney, but organ context determines whether a signal is adaptive, neutral or injurious (Li Z. et al., 2026). This context dependence argues against assigning a fixed beneficial or toxic label to a metabolite without dose, compartment and host-function information.

Several measurable signals recur across this axis: SCFAs, secondary bile acids, tryptophan-derived metabolites, LPS, IS, pCS and trimethylamine-N-oxide (TMAO) (Wang et al., 2020, 2023). In diabetic kidney disease, SCFAs have been linked to anti-inflammatory and renoprotective effects, whereas IS and pCS are associated with oxidative stress, inflammation and fibrosis (Kipp et al., 2025; Sun et al., 2026). In primary biliary cholangitis, multi-omics studies identify microbiota-metabolite patterns and bile acid profiles associated with clinical subtypes and prognosis (Han et al., 2024). In diabetic kidney disease, models integrating microbial and metabolite features can outperform traditional indicators (Chen et al., 2019). These examples show that cross-organ biology can be captured by combined microbiome and metabolomic readouts.

A systems approach is needed because these processes do not follow a single-organ or single-molecule logic. Serum creatinine and estimated glomerular filtration rate remain essential clinical indicators, but they have limited sensitivity for early CKD detection and mechanistic stratification (Peralta et al., 2011; Levin et al., 2024). Bidirectional metabolic, immune and transport feedback among gut, liver and kidney can also weaken single-point interventions (Gai et al., 2014; Wang et al., 2020). Future explanatory models should integrate metagenomics, metabolomics, transcriptomics and clinical phenotypes, then use machine learning or causal inference to move from correlation to testable mechanism.

## 6 Translational opportunities and research priorities

### 6.1 Biomarker and multi-omics strategies

Clinical readiness differs sharply across readouts. Serum creatinine, estimated glomerular filtration rate, albuminuria and established liver chemistry remain clinical tools, whereas strain-level microbial signatures, β-glucuronidase (GUS) or bile salt hydrolase (BSH) activity, fecal SCFAs and integrated metabolomic classifiers are predominantly research measures (Table 4). Translation requires reproducible pre-analytics, reference ranges, inter-laboratory harmonization, affordable assays and prospective incremental-value testing. Fecal markers are particularly limited because concentration reflects production, absorption, host use and transit and may not represent mucosal or systemic flux. Integrated models should therefore be compared against, rather than replace, established clinical predictors.

**Table 4:** Microbiota-derived readouts relevant to food xenobiotic exposure and functional interpretation.

Readout/target	Sample or assay	Food-xenobiotic interpretation	Functional significance	Current limitation	References
Microbial GUS activity	Feces; metagenome or enzymatic assay	Indicates capacity to deconjugate glucuronidated xenobiotics or endobiotics	May reactivate toxicants or drugs during enterohepatic cycling	Requires substrate-specific interpretation	Brossier et al. (2024)
Tryptophan-derived indoles	Feces; serum/plasma; urine	Link dietary protein/tryptophan intake with microbial AhR/PXR-active metabolites	Barrier integrity, immune tone and possible uremic toxin precursor pathways	Different indoles have opposite biological meanings	Venkatesh et al. (2014), Dodd et al. (2017), Graboski et al. (2023)
TMAO	Serum/plasma; urine	Reflect microbial conversion of choline/carnitine-rich foods to TMA and host oxidation	Cardiorenal and metabolic risk readout	Diet, renal function and host FMO activity confound interpretation	Romano et al. (2015), Rath et al. (2017)
SCFAs	Feces; serum/plasma	Reflect fermentation capacity and fiber-sensitive microbial function after dietary exposure	Barrier support, immune regulation and metabolic signaling	Fecal levels do not always reflect mucosal or systemic flux	Byndloss et al. (2017), van Dongen et al. (2021)
Indoxyl sulfate / p-cresyl sulfate	Serum/plasma; urine	Reflect microbial aromatic amino acid metabolism and kidney clearance	Protein-bound uremic toxins linked to oxidative stress and inflammation	More kidney-disease markers than general food-exposure biomarkers	Yu et al. (2011), Dou et al. (2015)
Bile acid profile	Serum/plasma; feces	Readout of microbial BSH and bile acid transformation after food or contaminant exposure	FXR/TGR5/PXR signaling, lipid metabolism and barrier function	Strong context dependence; disease and medication confounding common	Gai et al. (2014), Chen et al. (2019)
LPS, LBP and sCD14	Serum/plasma	Indirect readout of barrier disruption and metabolic endotoxemia after exposure	Systemic immune activation and inflammatory amplification	Low specificity; assay and pre-analytical variability	Cani et al. (2008), Wang et al. (2020)
BSH genes/activity	Feces; metagenome	Indicates bile acid deconjugation capacity relevant to diet, additives and contaminants	Modulates bile acid pool and FXR/FGF signaling	Gene presence does not guarantee functional flux	Begley et al. (2006), Jones et al. (2008)
Intestinal permeability markers	Serum/plasma; feces; functional tests	Potential readout of exposure-related barrier disruption	Translocation risk and inflammatory signaling	Commercial zonulin assays require caution and careful interpretation	Lammers et al. (2008), Ajamian et al. (2019)

AhR, aryl hydrocarbon receptor; BSH, bile salt hydrolase; FGF, fibroblast growth factor; FMO, flavin-containing monooxygenase; FXR, farnesoid X receptor; GUS, β-glucuronidase; LBP, lipopolysaccharide-binding protein; LPS, lipopolysaccharide; PXR, pregnane X receptor; sCD14, soluble CD14; SCFA, short-chain fatty acid; SCFAs, short-chain fatty acids; TGR5, Takeda G protein-coupled receptor 5; TMA, trimethylamine; TMAO, trimethylamine-N-oxide.

### 6.2 Dietary and microecological interventions

Intervention evidence is adjacent to, rather than direct proof of, food-xenobiotic risk reduction. CKD trials of probiotics, prebiotics, synbiotics and dietary restructuring are typically small and heterogeneous in strain or substrate, dose, duration, background diet, kidney stage and outcome definition. Meta-analyses report inconsistent effects across IS, pCS, inflammation and renal function (Yang H. -Y. et al., 2021). Future trials should use concealed allocation where feasible, matched controls, pre-specified exposure and metabolite endpoints, adequate duration, adherence measures and standardized adverse-event reporting. Safety requires particular attention in advanced CKD, cirrhosis, immunocompromise and other vulnerable states. These studies support hypothesis generation for microbial-metabolic modulation but not prevention claims for specific food-chain xenobiotics.

Precision nutrition should prioritize reproducible function over a single taxon. For example, a high proteolytic-fermentation or PBUT profile may support testing a fiber-rich, plant-forward pattern within kidney-specific potassium and protein constraints, whereas altered bile acid metabolism may justify evaluating fat quality and meal pattern rather than an isolated supplement (McFarlane et al., 2022; Gao et al., 2024). Implementation should proceed in stages: confirm the exposure and organ phenotype, measure a validated microbial or metabolite function, select a whole-diet intervention, and monitor both benefit and adverse effects. These examples are testable strategies, not current prescriptions based solely on microbiome composition.

### 6.3 Precision nutrition and microbial enzyme-targeting strategies

Targeting a microbial enzyme is conceptually different from suppressing the organism that carries it. Enzyme-selective inhibition may preserve community structure, but activity is often substrate-specific and the same enzyme family can participate in beneficial endobiotic pathways (Zhao et al., 2026). Microbial GUS inhibition currently provides the clearest proof of concept, mainly from drug reactivation models; azoreductases, nitroreductases and trimethylamine (TMA)-forming enzymes remain context-dependent candidate targets (Xu et al., 2026). The mycophenolate model demonstrates that a defined microbial reaction can be druggable, not that the same intervention reduces risk from food-derived compounds (Drevland et al., 2025). Off-target effects, compensatory pathways, altered enterohepatic cycling and long-term safety must therefore be tested for each substrate and host context.

The translational message is therefore threefold. Integrated microbiota-metabolomic models may improve risk stratification for liver, kidney and cross-organ disease. Probiotics, synbiotics and precision dietary strategies may reduce gut-derived toxin burden in selected settings. Directed inhibition of microbial enzymes is a longer-term possibility, but evidence specific to food-derived xenobiotics remains limited.

### 6.4 Future priorities: causality, standardization and clinical usability

The main bottleneck is causality. Priority designs include controlled feeding with measured contaminant content, repeated biospecimens that establish temporal order, isotope or targeted product tracing for microbial transformations, and interventions that perturb a defined strain, enzyme or metabolite while monitoring organ-relevant outcomes (González et al., 2023; Wei et al., 2023). Causal triangulation should combine mediation analysis, negative controls, gnotobiotic or ex vivo validation and, where valid genetic instruments exist, Mendelian randomization (Penning de Vries and Groenwold, 2023; Barznji, 2026). Sample size should be based on the expected exposure-by-microbiome interaction and corrected multi-omic testing rather than on taxonomic differences alone. Discovery models require pre-specified external validation across populations, dietary settings and analytical platforms before clinical or regulatory use.

Clinical use also requires standardization. Current consensus states that routine microbiome testing has limited evidence, and direct-to-consumer tests lack unified standards and confirmed clinical value (Porcari et al., 2025). The Strengthening The Organization and Reporting of Microbiome Studies checklist highlights the need to report sampling, sequencing, analysis and clinical metadata transparently (Mirzayi et al., 2021). Studies of dietary xenobiotics should adopt these standards from the design stage, including exposure measurement, annotation, batch-effect control and traceable reporting.

Timing is another unresolved issue. Cross-sectional designs often miss the sequence linking exposure, microbial metabolism and organ injury. Longitudinal multi-omics can better resolve dynamic changes in microbial communities and host states, although missing values, sparse signals and high dimensionality remain difficult (Sherwani et al., 2025). Integration frameworks such as MintTea show that joint analysis can identify multi-omic disease modules and generate mechanistic hypotheses (Muller et al., 2024). Future studies should combine repeated exposure measurement, sampling-window models and dynamic network analysis.

Single biomarkers are unlikely to capture liver-kidney network biology. In nephrology, bulk, single-cell and spatial omics can describe kidney biology more comprehensively, but data heterogeneity and platform differences remain major barriers (Saliba et al., 2024). In microbiome research, intermediate integration methods can link taxonomy, functional pathways and metabolomics into more robust disease-associated modules (Muller et al., 2024). Gut-liver-kidney studies should therefore prioritize joint modeling of metagenomics, metabolomics, host transcriptomics and clinical phenotypes.

Detectability is not the same as clinical utility. Consensus statements caution that broad routine microbiome testing is not yet evidence-supported, and many commercial tests lack clear indications or proven benefit (Porcari et al., 2025; Servetas et al., 2026). Translationally useful work should prospectively validate risk models that combine microbial features with exposure profiles, organ function, inflammation and outcome events (Leung et al., 2022; Porcari et al., 2025). The aim is not to produce more microbiome reports, but to build evidence chains that can guide dietary management, risk prediction and targeted intervention.

## 7 Future perspective

The evidence supports a food-chemistry-to-host-response model rather than a simple toxicity model. The same parent compound may be detoxified, remain largely unchanged or be converted into metabolites with different biological activity, depending on microbial enzyme capacity and host clearance. The gut-liver-kidney axis is therefore a useful organizing frame, but it should not be read as evidence that all dietary xenobiotics share one disease pathway.

Evidence quality varies by compound class and by the question being asked. Controlled human data are available for selected sweeteners and emulsifiers, whereas evidence for pesticides, plasticizers, PFAS, metals, mycotoxins and MPs is dominated by observational, animal or *in vitro* studies. Table 3 intentionally summarizes mechanistic food-exposure models and should not be read as a table of human chronic-disease risk. Direct biotransformation evidence is strongest when products or microbial genes are identified; dysbiosis and barrier associations occupy a lower causal tier. Human intervention evidence from CKD or drug toxicity is considered adjacent evidence and is not generalized to prevention of food-chain xenobiotic injury.

Risk assessment should therefore include both the parent exposure and the microbial transformation context. Current safety evaluations often focus on intake thresholds for single compounds. The evidence reviewed here suggests that dysbiosis, impaired barrier function, cholestatic or metabolic liver disease and declining kidney function may change the biological meaning of the same dietary exposure. These hypotheses require prospective testing before they can support individualized dietary advice.

This Review has clear boundaries. It does not establish population-level causality for chronic liver or kidney disease, and it does not argue that microbiome testing is ready for routine dietary risk management. The cited studies differ in exposure measurement, microbial profiling, outcome definition and control for diet or medication. High-dose animal models, acute exposure systems and fecal fermentation assays should be treated as mechanism-generating evidence. The most defensible conclusion is that microbial biotransformation modifies dietary xenobiotic effects, but is not yet a stand-alone clinical biomarker.

Future studies should trace exposure-metabolism-outcome chains. The most informative designs will combine repeated dietary and contaminant measurements, food processing information, strain-resolved metagenomics, targeted xenobiotic metabolite panels, host inflammatory or transcriptomic readouts and organ-specific endpoints. Reports should specify exposure source, dose, matrix and analytical method so that findings can be compared across studies. Intervention studies should first test whether modifying microbial enzymes or metabolites changes validated intermediate outcomes before claiming disease prevention.

## 8 Conclusion

Food-derived xenobiotics should be evaluated as food-borne chemical exposures whose effects are modified by microbial metabolism and host organ function. Gut microbes may detoxify selected compounds, bioactivate others or shift host responses through barrier injury, immune signaling, bile acid and tryptophan metabolism, SCFA depletion and uremic toxin generation. The gut-liver-kidney axis offers a useful map for organizing this evidence, but current data are stronger for mechanistic plausibility than for clinical prediction. Translation will require verified exposure data, microbiome function rather than taxonomy alone, metabolite confirmation and longitudinal organ-specific outcomes.
